# From emissions to incisions and beyond: the repercussions of climate change on surgical disease in low- and-middle-income countries

**DOI:** 10.1186/s12893-023-02260-8

**Published:** 2023-11-16

**Authors:** Russell Seth Martins, Kostantinos Poulikidis, Syed Shahzad Razi, M. Jawad Latif, Kyle Tafuri, Faiz Y. Bhora

**Affiliations:** 1 Division of Thoracic Surgery, Department of Surgery, Hackensack Meridian School of Medicine, Hackensack Meridian Health Network – Central Region, Edison, NJ 08820 United States of America; 2 Hackensack Meridian Health Network, Nutley, NJ 08820 United States of America

**Keywords:** Surgery, Environment, Sustainability, Healthcare, Operations, Equity, Global Surgery, Low- and middle-income countries

## Abstract

Climate change has far-reaching repercussions for surgical healthcare in low- and middle-income countries. Natural disasters cause injuries and infrastructural damage, while air pollution and global warming may increase surgical disease and predispose to worse outcomes. Socioeconomic ramifications further strain healthcare systems, highlighting the need for integrated climate and healthcare policies.

Climate change is termed the biggest health threat of the 21st century and also features prominently in the United Nation’s Sustainable Development Goals. Ironically, the healthcare sector itself is a contributor to climate change. It has been estimated that the health sectors of the United States, Australia, England, and Canada (all high-income countries) emit more greenhouse gases than all but six countries worldwide [1]. In low- and middle-income countries (LMICs), where over 90% of the population lack access to safe and affordable surgical care [2], climate change acts as a “multiplier” to exacerbate existing healthcare problems and create new ones. The impact of climate change on surgical healthcare in these countries is a significant concern, with implications for both patients and healthcare systems.

## The acute: trauma, injuries, and infrastructural damage

Climate disasters have become five times more frequent over the last 50 years. Over this period, weather disasters have accounted for approximately 2 million deaths globally, over 90% of which occurred in LMICs [2]. Approximately 7 million surgical procedures are performed annually for natural disaster-related injuries, with this number expected to increase significantly and disproportionately in LMICs. LMICs are ill-equipped to deal with the additional burden of emergent traumatic or physical injuries associated with climate disasters, due to limited infrastructure and resources, healthcare workforce shortages, and transportation and geographic barriers. In Pakistan, where there is one surgeon per approximately 140,000 persons for a population of over 220 million, flooding in 2022 led to over 1,700 deaths and 13,000 traumatic injuries, and damaged over 1,460 healthcare centers across the country. These floods also disrupted obstetric care, with approximately 2,000 women a day giving birth mostly in unsafe conditions. Even before the floods, Pakistan had one of the highest maternal mortality rates in Asia. The case of Pakistan, described above, is but one example of the devastating impacts that natural disasters can have on surgery and allied healthcare.

Apart from natural disasters, a landmark study in 2022 tied increasing ambient temperatures to a growth in interpersonal gun violence. While this phenomenon has not been validated in other settings, countries with greater income inequality have higher rates of criminal violence and conflict and may be at an increased risk due to the social, economic and political repercussions of climate change [3].

## The insidious: surgical disease and surgical outcomes

Air pollution is recognized as a risk factor for lung cancer [4], with over 16% of lung cancer cases globally and 40% of cases in East Asia being attributable to air pollution. There is also an increasing body of literature tying environmental pollution to the development of other cancers, such as breast and bladder cancer [5]. Infections with surgical indications (e.g., trachoma, filariasis, fungal and parasitic infections, and ocular and skin infections) may also be increased by warmer temperatures, excess rains, and flooding [6]. Extreme temperature events, air pollution, and even chronic stress brought on by serious natural disasters, may increase the risk or mortality associated with acute myocardial infarctions [7, 8].

Climate change also causes adverse obstetric and maternofetal outcomes through mediating factors such as increased malnutrition, infections, population displacement, and higher ambient temperatures. Air pollution and extreme heat increases the risk of preterm birth and low birthweight, which predisposes newborns to disease requiring urgent surgical management such as necrotizing enterocolitis. Maternal heat exposure has also been linked to an increased risk of congenital heart disease [9]. Even wildfires, which receive much media attention in HICs but are by far more prevalent in LMICs, may contribute to adverse fetomaternal outcomes [10]. Given that 99% of the maternal and neonatal mortality occurs in LMICs and that over 90% of air pollution-related deaths occur in LMICs [11], it is clear that these under-resourced regions are particularly vulnerable to the maternofetal impacts of climate change.

Climate change may also have implications for surgical outcomes. Warmer temperatures increase the incidence of surgical site infections (which have a greater incidence in LMICs compared to HICs) by up to 39% [12], while ambient air pollution has been shown to increase hospital length of stay, healthcare costs, and readmission rates [13]. Air pollution can also significantly impair recovery of lung function and even increase mortality after lobectomy for lung cancer [14].

## The over-arching: society, economics, and politics

Climate change also poses complex challenges to the socioeconomic and political fabric of LMICs. These may produce ripple effects on surgical healthcare. Natural disasters or changes in rainfall patterns can drastically impact agriculture and water availability, leading to food insecurity. Malnutrition impacts approximately one-third of the population living in LMICs [15] and is a known risk factor for adverse outcomes after surgery. Decreased agricultural productivity also has major financial ramifications for LMICs, which may also suffer significant financial losses in tourism and extractive industries. Economic instability limits investment in improving access to surgical services, medical technology, workforce, and surgical research and innovation. Natural disasters can also increase population displacement and migration with LMICs, which can strain already overburden already scarce resources. Language and cultural barriers, as well as the psychosocial comorbidity associated with being displaced, can also impact the surgical experience amongst displaced populations. Climate disasters can also significantly impact education in LMICs, including medical education and training, which can have downstream effects on the future surgical workforce.

Environmental policy can be a point of political cooperation or polarization. Likewise, political philosophies on key aspects of surgical healthcare also vary widely, including type of coverage, financing, role of the private sector, public health and preventive healthcare, and regulatory frameworks. Effective and bold political leadership is needed to achieve synergy between environmental and healthcare policy to strengthen healthcare systems, enhance disaster preparedness, and target equitable access to healthcare globally.

## Operation sustainability: surgeons at the forefront

The surgical community must recognize its responsibility and its unique opportunity to play a pivotal role in mitigating the impact of climate change within their field. Some actionable areas are as follows:

- Adopt environmentally sustainable practices within their surgical practice and work towards decreasing the environmental footprint of surgical healthcare in general (Table 1).
- Work with public health professionals to design and implement climate crisis management strategies, ensure adequate surgical supplies, and provide disaster-management training to surgical staff to reduce surgical morbidity & mortality associated with climate disasters.
- Raise awareness amongst colleagues, policymakers, and the public regarding the need for policies and initiatives that address climate change and its repercussions on healthcare.
- Champion research that explores the relationship between climate change and surgical disease so as to better anticipate, prepare for, and even mitigate potential challenges.
- Collaborate with all other stakeholders involved, including other healthcare professionals, researchers, administrators, policymakers, sustainability experts, and politicians.
- Incorporate climate change and its effects on surgical diseases and healthcare into medical education and surgical training programs. Equipping future surgeons with the knowledge and skills to manage the challenges posed by climate change can ensure continuity of efforts and groom future leaders in this space.

In conclusion, our evolving understanding of the repercussions of climate change on surgical disease and healthcare underscores the need for urgent, collaborative, multi-dimensional action to safeguard global wellbeing for the future.

**Table 1:** Multi-level strategies for the surgical community to become more climate conscious and adaptable [16]

Aspect of Surgery	Possible Strategies for Increased Climate Consciousness
Surgical Healthcare Provision	• Green anesthetic practices: o Reducing desflurane use, reducing nitrous oxide use. o Using low-flow settings when using volatile anesthetics. o Increase use of intravenous anesthesia • Green operative practices: o Reducing plastic syringe use o Reducing unnecessary intravenous fluid and drug use o Using reusable surgical gowns & drapes o Using reusable surgical equipment, including laparoscopy ports • Green operating theatre practices: o Turning off lights when not in use o Turning off heating/cooling systems at nights and on weekends o Installing energy-efficient lighting o Turning off taps between hand washing • Telemedicine and remote consultations
Surgical Education & Training	• Incorporating sustainability curriculum in education and training • Virtual education
Surgical Health Systems	• Design and construct healthcare facilities using sustainable building practices, energy-efficient systems, and renewable energy resources • Improving waste management programs • Optimize sterilization processes for water and energy use • Sustainable supply chains
Surgical Academia	• Promote research on the intersection between surgery and climate change through strategies such as dedicated scientific journals, journal special issues, and scientific conferences
Surgical Innovations	• Designing surgical devices with a lower environmental footprint

## Data Availability

Not relevant/not required.

## Abbreviations

*LMICs*: Low- and middle-income countries
